# Defining the *Helicobacter pylori* Disease-Specific Antigenic Repertoire

**DOI:** 10.3389/fmicb.2020.01551

**Published:** 2020-07-09

**Authors:** Maria Felicia Soluri, Simone Puccio, Giada Caredda, Paolo Edomi, Mario Milco D’Elios, Fabio Cianchi, Arianna Troilo, Claudio Santoro, Daniele Sblattero, Clelia Peano

**Affiliations:** ^1^Department of Health Sciences & IRCAD, Università del Piemonte Orientale, Novara, Italy; ^2^Center for Translational Research on Autoimmune and Allergic Disease, Università del Piemonte Orientale, Novara, Italy; ^3^Laboratory of Translational Immunology, IRCCS, Humanitas Clinical and Research Center, Rozzano, Italy; ^4^Department of Excellence in Pharmacological and Biomolecular Sciences, University of Milan, Milan, Italy; ^5^Department of Life Sciences, University of Trieste, Trieste, Italy; ^6^Department of Experimental and Clinical Medicine, School of Human Health Sciences, University of Florence, Florence, Italy; ^7^Institute of Genetic and Biomedical Research, UoS Milan, National Research Council, Milan, Italy; ^8^Genomic Unit, IRCCS, Humanitas Clinical and Research Center, Milan, Italy

**Keywords:** *H. pylori* infection, interactome, gastric cancer, MALT lymphoma, autoimmune gastritis, phage display, next generation sequencing

## Abstract

The analysis of the interaction between *Helicobacter pylori* (HP) and the host *in vivo* is an extremely informative way to enlighten the molecular mechanisms behind the persistency/latency of the bacterium as well as in the progression of the infection. An important source of information is represented by circulating antibodies targeting the bacteria that define a specific “disease signature” with prospective diagnostic implications. The diagnosis of some of the HP induced diseases such as gastric cancer (GC), MALT lymphoma (MALT), and autoimmune gastritis (AIG) is not easy because patients do not show symptoms of illness in early-onset stages, at the same time they progress rapidly. The possibility of identifying markers able to provide an early diagnosis would be extremely beneficial since a late diagnosis results in a delay in undergoing active therapy and reduces the survival rate of patients. With the aim to identify the HP antigens recognized during the host immune-response to the infection and possibly disease progression, we applied a discovery-driven approach, that combines “phage display” and deep sequencing. The procedure is based on the selection of ORF phage libraries, specifically generated from the pathogen’s genome, with sera antibodies from patients with different HP-related diseases. To this end two phage display libraries have been constructed starting from genomic DNA from the reference HP 26695 and the pathogenic HP B128 strains; libraries were filtered for ORFs by using an ORF selection vector developed by our group (Di Niro et al., 2005; Soluri et al., 2018), selected with antibodies from patients affected by GC, MALT, and AIG and putative HP antigens/epitopes were identified after Sequencing and ranking. The results show that individual selection significantly reduced the library diversity and comparison of individual ranks for each condition allowed us to highlight a pattern of putative antigens specific for the different pathological outcomes or common for all of them. Within the putative antigens enriched after selection, we have validated protein CagY/Cag7 by ELISA assay as a marker of HP infection and progression. Overall, we have defined HP antigenic repertoire and identified a panel of putative specific antigens/epitopes for three different HP infection pathological outcomes that could be validated in the next future.

## Introduction

*Helicobacter pylori* (HP) infects more than 50% of the world’s population (Parsonnet, 1995; Goh et al., 2011; Peleteiro et al., 2014; Hooi et al., 2017) thus being the most prevalent human pathogen worldwide. Infection occurs in early childhood and colonization most likely persists for the long-life. The bacterium colonizes the stomach, not only residing on the surface area of the gastric mucosa but also adhering to the gastric epithelium (Stolte and Eidt, 1989; Björkholm et al., 2000). Although in most people the infection remains clinically asymptomatic, most colonized individuals develop coexisting chronic inflammation that significantly increases the risk of site-specific diseases. Many studies have demonstrated that HP infection causes gastric and duodenal ulcer diseases, that can lead to Gastric Cancer (GC) and mucosa-associated lymphoid tissue (MALT) B-cell lymphoma (Wyatt and Rathbone, 1988; Shahar et al., 1994). Several factors act, alone or synergistically, during the infection, and their combination can determine the fate of the infection progression, making the difference between latent infection and the development of a pathological outcome. Dysregulation of the inflammatory/immune host response plays an important role in the progression toward cancer. A genetically determined pro-inflammatory state increases the risk of cancer, and several human polymorphisms occurring within genes of the inflammatory/immune-response have been associated with gastric malignancies development (El-Omar et al., 2000; Yuzhalin, 2011). Among bacterial components, some factors associated with malignancy have been also identified, although the high grade of genomic variability of HP strains has hindered this purpose. The most important virulence factor of HP is the cag pathogenicity island (cagPAI), a genetic locus of about 40 kb that contains 31 genes (Tomb et al., 1997; Alm et al., 1999) and encodes for the so-called type IV secretion system (T4SS). This forms a syringe-like structure that injects bacterial components (mainly peptidoglycan and the oncoprotein cagA) into the host target cell (Rohde et al., 2003). HP strains that harbor the cagPAI (cagPAI+) pathogenicity locus show a significantly increased ability to induce severe pathological outcomes in infected individuals, compared to cagPAI− strains (Blaser et al., 1995; Kuipers et al., 1995; Noto and Peek, 2012; Takahashi-Kanemitsu et al., 2020).

Current treatments of HP infection are based on double-antibiotic therapy combined with proton-pump inhibitors. However, this therapeutic approach is not optimal, not only for its many drawbacks but also for the insurgence of antibiotic resistances and its consequent loss of efficacy (Thung et al., 2016; Hu et al., 2017). Furthermore, this strategy of eradication, even when successful, does not lead to any protection from subsequent re-infections (Solnick, 1998; Megraud et al., 2013). The development of a vaccine has been a field of research pursued in the last decades. Progress have been made, and different antigens, adjuvants, administration routes, have been explored, leading to promising results, either using prophylactic and therapeutic protocols, in animal models; however, the gaining of protective immunity in humans remains elusive (Aebischer et al., 2008; Müller and Solnick, 2011; Salama et al., 2013; Ikuse et al., 2019). A deeper understanding of host-bacterium interaction is needed. However, this goal is limited by the fact that many molecular aspects of the bacterium are still incompletely characterized, with about 23% of the bacterium proteins that remain without a defined functional annotation (Tomb et al., 1997; Boneca et al., 2003; Resende et al., 2013). Identification and characterization of novel predictive biomarkers could also improve the power of serological tests for detecting and monitoring HP infection and could be beneficial for both therapeutic and diagnostic purposes. HP related GC detection is currently based on the use of markers prone to a high degree of false-negative results, thus making the discovery of novel sensitive biomarkers an urgent issue (Lam and Lo, 2008). Moreover, there is an important need to improve earlier ability to identify patients that are progressing toward cancer development, to target those patients and make them undergo early clinical programs for cancer prevention.

In this study, we aimed to (i) construct a genomic library of *H. pylori* to be representative of all the ORFs of the bacterium, and to (ii) identify novel HP antigens recognized by host immune system response. We applied an unbiased approach based on the production of phage libraries for the displaying of the whole antigenic repertoire of two HP strains. This approach allowed the identification of different antigenic domains of *H. pylori*. Among these we focused our attention on the protein CagY/Cag7. Here we report and characterize, the presence of antibodies against a domain of the CagY protein, in a high percentage of patients affected by different pathological outcomes related to HP infection.

## Materials and Methods

### Bacterial Strains

Two *H. pylori* strains have been used for ORF/filtering libraries preparation: HP 26695 *H. pylori* strain described by Tomb et al. (1997); it was originally isolated from a patient suffering gastritis and shown to be able to elicit immune and inflammatory responses and is considered the reference strain. HP B128(B8) *H. pylori* strain more recently described and isolated from a patient with a gastric ulcer (Israel et al., 2001; McClain et al., 2009).

*Escherichia coli* DH5αF’ (Gibco BRL), F’/endA1 hsd17 (rK− mK +) supE44 thi-1recA1 gyrA (Nalr) relA1 (lacZYA-argF) U169 deoR [F80dlacD-(lacZ)M15], were used for both libraries preparation and phage display selection.

### Human Sera Samples

Patients’ sera have been collected at the Careggi Hospital in Florence in collaboration with the Department of Experimental and Clinical Medicine of the University of Florence; the recruitment of patients has been approved by the Ethical committee protocol number 14936_bio. Autoimmune Gastritis (AIG) patients: all patients with AIG had both autoantibodies against parietal cells (APCA) and intrinsic factor (AIFA) (D’elios et al., 2001; Troilo et al., 2019). Histology is considered the most reliable method for assessing the presence of AIG (Massironi et al., 2019). Histopathological assessment of gastric mucosa was scored according to the Sydney System classification (Dixon et al., 1996) on a visual analog scale (0 = absent, 1 = mild, 2 = moderate, 3 = severe). Six patients were classified at stage II; four patients were classified at stage III. Patients with GC: all patients studied had distal gastric adenocarcinoma. Each patient was carefully clinically evaluated, and the TNM stage recorded. Seven patients were classified with a TNM stage I; 16 patients with TNM stage II; six patients with TNM stage III. Patients with gastric MALT lymphoma: six newly diagnosed MALT lymphoma patients, classified as Ann Arbor stage, were enrolled in this study. Five patients were classified at stage I, only one patient was classified at stage II. Data relative to the classification and stage of the patients have been included in Supplementary Table 3. IgG antibodies were purified from all sera samples by affinity chromatography on protein A agarose (Thermo Fisher Scientific). Single sera were diluted in PBS and incubated with protein agarose for 16 h at 4°C with gentle rotations. Bound antibodies were eluted with 0.1 M Glycine pH 2.7 and immediately buffered with 20% vol/vol of 1M Tris–HCl pH 8.5. Following dialysis against PBS, purified antibodies were quantified on SDS-PAGE and Coomassie staining.

### Genomic ORF Libraries Preparation

*Helicobacter pylori* ORFeome-libraries were prepared in the pFILTER3 vector, as previously described (Zacchi et al., 2003; D’Angelo et al., 2011; Soluri et al., 2018). Briefly, the genomic DNA from the three different strains were separately fragmented by ultra-sonication (Covaris) (duty cycle 10%; intensity 5.0; cycles per burst 200; duration 2 × 60 s, total 120 s; mode frequency sweeping; temperature 6°C) to obtain fragments ranging from 200 to 800 bp length. Fragments were treated with the Quick blunting kit (New England Biolabs) to perform end-repairing and phosphorylation and then ligated into the vector previously blunt-cut through *Eco*RV enzyme digestion (New England Biolabs). Transformation of the two libraries obtained into DH5αF’ bacteria cells was performed through electroporation. After electroporation, bacteria were plated on 2×TY agar plates supplemented with 34 μg/ml chloramphenicol (pFILTER resistance) and 25 μg/ml ampicillin (selective marker for ORFs) and grown for 16 h at 37°C. Dilutions of each library were also plated and grown in parallel to determine the library size and efficiency of the filtering. Bacteria were harvested, thoroughly mixed and stored at −80°C in 20% sterile glycerol, in small aliquots. Plasmid DNA was extracted from one aliquot and used to prepare the phagemid libraries. To this aim filtered ORFs were cut from each pFILTER library with *Bss*HII and *Nhe*I restriction enzymes (New England Biolabs), and cloned into a compatible pDAN5 vector (Sblattero and Bradbury, 2000) upstream of the g3p gene. Two different libraries, one for each HP strain, were prepared. Libraries were titrated and analyzed by deep sequencing.

### Library Selection Procedure

Four pools of sera were prepared mixing equal amount of purified antibodies derived from three to six donors. Phage particles preparation and selection procedures were done according to established protocols (Di Niro et al., 2009; Dal Ferro et al., 2019). Briefly, phages were diluted in MPBS (2% non-fat milk in PBS), added to 30 μl of DynaBeads ProteinA (Life Technologies) with 10 μg of antibodies immobilized on, incubated for 90 min at room temperature in rotation. Beads were washed five times with PBS-0.05% Tween-20 and five times with PBS, and then phages were eluted by the addition of 1 ml DH5αF’ at OD_600 nm_ = 0.5 for 45 min with occasional shaking. Bacteria obtained after the first cycle of selection were amplified and phages prepared for a second cycle where the washing conditions were modified to improve specificity. In the second cycle after incubation with the phages, beads were subjected to 10 times washes with PBS-0.1% Tween-20, then resuspended in PBS and incubated for 10 min on rotation and washed again for 10 times with PBS. Bacteria cells used for the elution were plated on 2×TY agar plates supplemented with 100 μg/ml of ampicillin and grown for 16 h at 37°C. The outputs of each selection were collected, thoroughly mixed and stored at −80°C with 20% sterile glycerol. Plasmid DNA was extracted and analyzed by deep sequencing.

### Illumina Sequencing and Data Analysis

The following protocol has been used to perform recovery, sequencing and analysis of DNA inserts from pFILTER-ORF-library, pDAN5-ORF-library or selected-phage-libraries. DNA inserts were recovered by a first PCR amplification with specific primers annealing on the plasmid backbone. These primers carry at their 5′ end specific adaptors sequences for the successive indexing and the direct sequencing of the amplicons (primers sequences and protocol details are explained in Soluri et al., 2018). After the first PCR clean-up, a second Index PCR was performed by using the Nextera XT Index kit (Illumina) to produce double indexed amplicon pool. Libraries have been qualitatively evaluated on the 2200 TapeStation (Agilent) to verify their size and in parallel quantified by Real Time PCR by using the KAPA Biosystem library quantification kit (Roche) following manufacturer’s protocol. Libraries were sequenced by generating long reads, at least 250 bp Paired End by using the MiSeq Illumina instrument. Bioinformatic analysis was performed by using the InteractomeSeq webtool (Puccio et al., 2020) with a pipeline specifically developed by our group for the analysis of this kind of data. In brief the data analysis workflow allows generating a list of putative domains with genomic annotations just starting from raw sequencing reads. The pipeline is composed of four sequential steps: (1) input files are checked (raw reads, reference genome sequence, annotation list) to verify their proper format; (2) low-quality sequencing data or reads with a length lower than 100 base are trimmed using Cutadapt (Martin, 2011); (3) reads are aligned with blastn (Madden, 2013) to the genome sequence allowing up to 5% of mismatches, and a SAM file is generated. Only reads with quality score greater than 30 (Q > 30) are further subjected to SAMtools (Li et al., 2009) processing and converted into a BAM file; reads falling into a CDSs for at least 80% of their length are identified by invoking BEDTOOLS (Quinlan and Hall, 2010); then, for each CDS portion covered by mapping reads, the analysis pipeline calculate and take into account gene coverage (the total number of reads assigned to a gene), max depth (the maximum number of reads covering a specific genic portion) and the focus index (obtained from the ratio between max depth and coverage). CDS portions showing a focus index higher than 0.8 and a coverage higher than the average coverage observed for all mapping regions in the BAM file are classified as putative domain/epitope. (4) Finally, a list of putative domains is generated in tabular separated format and their relative tracks that can be viewed in the genome browser. Comparison between common and specific domains enriched in different selection experiments can be performed and Venn diagrams are generated. Raw sequencing Data available at Sequence Read Archive under Bioproject Accession number PRJNA506709.

### Cloning and Expression of Recombinant Protein

*Helicobacter pylori* HP0527 ORF corresponding to aa 566–655 was chosen for validation. The phage expressing the selected fragment was recovered from the phagemid library thanks to two back-to-back oligonucleotides mapping to the center of the enriched domain identified by the overlapping reads. ORF coding fragment was then excised from the phagemid vector and cloned into a compatible pGEX-4T1 (GE-Healthcare) vector for the expression of the selected ORF as GST-fusion product. The successful cloning of the specific gene fragment was assessed by sequencing analysis. Positive colonies were grown until OD_600 nm_ = 0.5 and induced by IPTG with a final concentration of 0.2 mM for 16 h at 28°C. The recombinant protein was purified with GST affinity resin (Sigma-Aldrich). Purity and integrity of purified GST-ORF protein domain were checked by SDS-PAGE followed by Coomassie gel staining. Concentration was assessed by densitometry using ImageJ analysis software (Wayne Rasband, NIH, United States^1^) and calculated based on a standard BSA curve. This analysis was paralleled by measure the OD at 280 nm of the protein preparation. The purified protein was also checked by western blotting with α-GST and α-FLAG antibody.

### ELISA Assay

An ELISA assay was set up to validate the antigenicity of the selected HP0527 protein portion. Briefly, 96 plate ELISA wells (Greiner) were coated by an overnight incubation at 4°C with 200 ng/well of recombinant GST-protein diluted in PBS. After washing with PBS, wells were blocked with 200 μl of blocking solution (PBS-0.05% Tween-20-2% milk) through incubation at 37°C for 1 h. Wells were again washed for three times with PBS-0.05% Tween-20 and incubated with sera samples diluted 1:500 in blocking solution, for 2 h at 37°C. After five washes with PBS-0.05% Tween-20 and five washes with PBS, wells were incubated for 1 h at 37°C with a goat α-human IgG HRP conjugated (Sigma-Aldrich) diluted 1:5000 in blocking solution. Wells were washed again, immune-complexes revealed with TMB and the plate read at 450 nm. Samples with absorbance ≥ of the mean OD450 value obtained with healthy control sera plus 2 standard deviations (SD) were considered positive.

### Statistical Analysis

ELISA results were analyzed by using GraphPad Prism 8 software and differences between groups evaluated by the Mann–Whitney test; a *p*-values < 0.05 was considered statistically significant.

## Results

### Construction of *Helicobacter pylori* Phage Display ORFs Library

We have developed a pipeline for the identification and validation of novel antigenic proteins in different pathological conditions (D’Angelo et al., 2013; Antony et al., 2019). The approach combines the use of ORF filtering to select folded protein domains, phage display system to select the domain of interest and the power of deep sequencing technology for the identification of enriched clones. This procedure has been shown to be feasible for making and selecting libraries from bacteria whole genomes (D’Angelo et al., 2011; Gourlay et al., 2015) therefore we applied it to identify novel HP antigenic proteins.

Two HP strains were initially selected: the HP 26695, considered the reference strain, and originally isolated in the United Kingdom from a patient suffering from gastritis, and HP B128, isolated in the United States from a patient with a gastric ulcer. By using genomic DNA from both strains, ORF filtered libraries were individually produced. The genomic DNA was extracted and randomly fragmented in the range of 200–800 bp; this length range should provide a broad representation of the whole “domainome” of the bacterium (Kuznetsov et al., 2006). gDNA fragments were end-repaired and cloned into the filtering vector in which only folded ORF fragments can allow transformant *E. coli* cells to survive under selective pressure. After the filtering procedure the size of the libraries was reduced to 1/50 respect to the non-filtered ones, with a final size being 1.4 × 10^6^ and 1 × 10^6^ colonies for HP 26695 and HP B128, respectively. This selection rate was in keeping with theoretical calculation as well as our previous observations (Zacchi et al., 2003; D’Angelo et al., 2011) suggesting successful ORF filtration. Fragment size variability was assessed by PCR on random clones showing an average length of 150–200 bp. Considering the size of the starting *H. pylori* genome the libraries provide coverage of around 100-fold. After the filtering step, the ORF DNA fragments were subcloned into a phage display vector to allow their expression as a fusion protein with the g3p protein. The final library size was 1 × 10^6^ clones per each strain.

### ORF-Filtered Genomic Libraries Characterization by Deep Sequencing

The phage display libraries were analyzed by deep sequencing. Plasmid DNA was extracted from each library and ORFs were amplified, sequenced and analyzed to determine whether they were representative of the whole ORFeome of the two HP strains. More than 1.4 million and 1 million reads were produced for HP 26695 and HP B128 phage libraries respectively, thus reaching a total genome sequencing depth higher than 76X and 77X for HP 26695 and HP B128. Considering the coverage of the known CDSs of both strains we found that more than 93% of the total CDSs for both HP 26695 and of HP B128 where represented in their respective ORF libraries. When looking at the percentage of nucleotide covered by the reads inside CDSs, this was respectively, 73.5 and 76.8% (Table 1) for HP 26695 and of HP B128. In total we identified 1372 CDS domains represented into the HP 26695 phage library and 1597 CDS domains in the HP B128 phage library. It should be noted that the number of domains detected can be higher than the number of total CDSs in the genome because more than one domain can be found inside a single CDS.

**TABLE 1:** Sequencing and mapping metrics.

	HP 26695	HP B128	Control	SelA	SelB	SelC
Raw reads	1,425,554	1,031,956	2,169,178	815,891	3,737,010	1,633,626
Reads after trimming	1,216,124	954,895	1,798,722	680,764	3,149,939	1,278,335
Mapping Reads	1,114,184	746,325	1,294,576	503,401	2,563,391	961,686
% of Mapping reads	91.61%	89.10%	71.97%	73.94%	81.37%	75.22%
Unmapping reads	11,158	158,301	166,831	113,397	257,481	128,346
Mean coverage	76.81X	76.81X	96.13X	31.19X	155.82X	56.97X
CDS covered	1372	1597	1225	1130	1331	1263
% CDS covered	93.46%	93.80%	83.44%	76.97%	90.66%	86.03%
Nucleotides covered inside CDS	1,068,020	1,164,096	703,739	898,694	755,447	653,331
% of Nucleotide covered inside CDS	73.51%	76.80%	48.44%	61.86%	52.01%	44.97%

### Comparison of the Two ORF-Filtered Libraries

After the initial characterization, we tried to understand if the libraries obtained from the two HP strains, had a high level of similarity, thus we performed a mapping comparison by aligning the reads obtained from the sequencing of the two genomic ORF-filtered libraries of HP 26695 and of HP B128 against the reference genomic sequence of HP 26695 (accession number NC_000915). After mapping 91.61% of the trimmed reads of HP 26695 library aligned with its own reference genome, at the same time we observed that 89.10% of trimmed reads of HP B128 library aligned with the genome sequence of HP 26695 (Table 1). After this analysis, we concluded that the genetic diversity that separates these two strains does not significantly affect the nature of ORFs/domains sequences filtered out. In the light of these results, we decided to use the HP 26695 as the reference genome and to use its CDSs annotation to functionally read out the results deriving after the selection step performed with sera from patients. At this point to understand if the common ORFs/domains, represented inside the two genomic phage libraries could have the same features, in terms of the portions of the CDS filtered out, we compared their amino acidic sequences by blasting all the HP 26695 protein domains against all the HP B128 protein domains using BLASTX (search protein databases using a translated nucleotide query). The blasting step was performed by imposing the following parameters: amino acidic sequence identity >30% and length overlapping >50%. Among the 1268 common domains 98.82% have an amino acid sequence identity higher than 30%. Thus, the overlapping between the two genomic ORF filtering libraries was confirmed also from a functional point of view.

### Selection of Phage ORFs Library With HP Patient’s Sera

Based on the previous results we decided to pool the two ORFs library and use them as a single reagent in order to maximize the diversity of functional domains displayed on phage.

We focused on three different HP related diseases: GC, AIG, and MALT lymphoma. For each of these diseases we prepared a pool of sera, using affinity-purified Igs, to be used for the selection of the library: pool A, was made by six patients who developed GC (Selection A); Pool B, from four patients who developed AIG (Selection B) and Pool C from three patients who developed MALT lymphoma (Selection C). Finally, as control we also prepared a pool from healthy donors that were HP positive (Control Selection). The pooling of sera was applied as it was previously shown (D’Angelo et al., 2013) to be successful in reducing the inter-individual variability of antibody titer.

Each of the four pools of sera was independently used as a bait to select the pooled ORF phage display library. Before each round of selection, to reduce unspecific binding, a clearing selection step was introduced by incubating phages with magnetic beads pre-coated with a recombinant construct carrying the Fc portion of human IgG. Two consecutive cycles of selection and amplification were performed, increasing stringency of washing and binding conditions in the second cycle.

### Selected Libraries Characterization by Deep Sequencing

**FIGURE 1:**
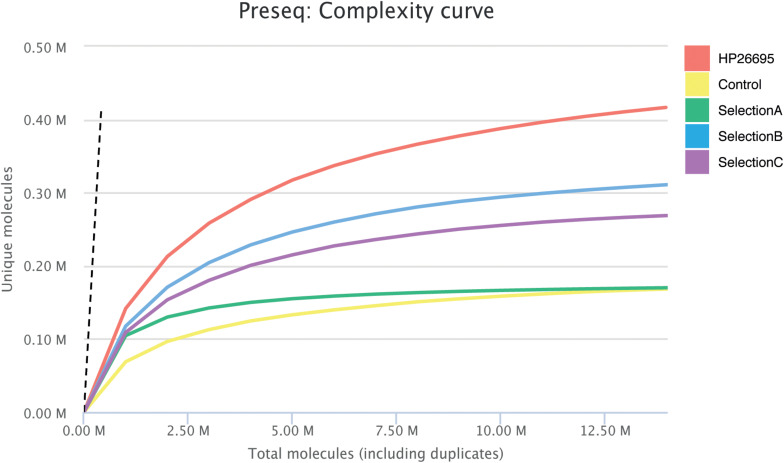
Estimation of Library complexity. Plotted values generated by Preseq showing estimated number of distinct reads for the Genomic Phage Library and for Phage Selections with mean values for ≤10 million reads. The estimates were used to examine and optimize the sequencing depth, and to avoid low complexity samples. The dashed line shows a perfectly complex library where total reads = unique reads.

After the second cycle of selection DNA coding for ORF fragments was amplified and sequenced. In total, more than nine million paired-end reads were produced by sequencing the four different selections. We generated 2,169,178 reads for the Control-Selection library (healthy controls), 815,891 reads for the Selection A (GC-sera), 3,737,010 reads for Selection B (AIG-sera) and 1,633,626 reads for Selection C (MALT-sera). After the alignment step it resulted that from 72 up to 81% of the reads were mapped against the reference genome (Table 1). When analyzing the CDSs present in the selected libraries we counted only the ones being represented by a minimum of ten reads, the total number of HP CDSs represented in the four selection libraries was 1225, 1130, 1331, 1263, respectively (Table 1). Interestingly the number of CDS detected after the selection was comparable with that presented in the starting library nevertheless the portion of CDSs covered by the selected sequences decreased to 48.44% (Control Selection), 61.86% (Selection A), 52.01% (Selection B) and 44.97% (Selection C) (Table 1). The reduction of the ORF library coverage after selection with sera is clearly represented by the Preseq plot in Figure 1 that shows a progressive reduction of the complexity curve from the genomic phage library that shows the maximum variability up to the Healthy Control phage library that has the lowest complexity. This result suggests that enrichment of specific portions of CDSs/Domains likely occurred after selection with the patient’s sera.

### Ranking of Enriched Domains/Epitopes

**FIGURE 2:**
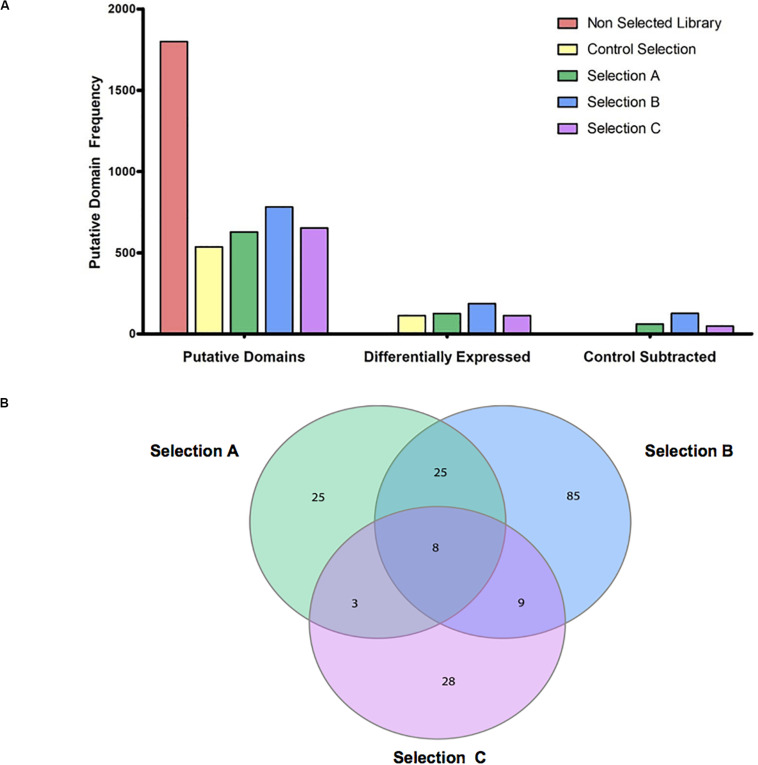
Overview of putative domains detected, enriched and specific for selections. **(A)** The bar group on the left shows the total amount of Putative domains that are detected for HP 26695 ORF-filtering library (Non-selected Library), and the selections with the antibodies from healthy control subjects (Control Selection) and from patients with the three pathological outcomes (Selection A-Gastric Cancer, Selection B-Autoimmune Gastritis, and Selection C-MALT lymphoma); the bar group in the middle shows the total number of domains resulting as enriched in the four selections compared to the Genomic HP 26695 ORF-filtering library; the bar group on the right side of the graph shows the numbers of domains detected in the Selection A, B, and C outputs after the exclusion of those domains in common with the Control Selection. **(B)** Venn diagram of specific and common putative antigens. Domains enriched in the Selection A, B, and C are indicated in green, blue, and pink, respectively.

The identified domains in the four libraries were ranked according to three consecutive steps of analysis. Initially, we counted the putative epitopes that were enriched and identified in the Control Selection (Healthy Control-sera) and in the Selection A (GC-sera), Selection B (AIG-sera), and Selection C (MALT-sera) these being 535, 627, 780, and 652, respectively (Figure 2A) (the list of all the annotated domains is reported in Supplementary Table 1 and the complete nucleotide and amino acids sequences is reported in the Supplementary File). Among the top proteins the more recurrent in all the three selections with sera from patients (Sel A, Sel B, Sel C) and in the selection with the sera from healthy controls, worth of note are: HP0527 cag pathogenicity island protein Cag7 (CagY), HP0459 protein VirB4 and HP1341 siderophore-mediated iron transport protein TonB. Among these top represented and positively recognized proteins the only one that is specifically listed only in the patients’ sera selections (Sel A, Sel B, Sel C) is HP0527 cag pathogenicity island protein Cag7 (CagY), as a matter of fact, both HP0459 protein VirB4 and HP1341 siderophore-mediated iron transport protein TonB are recognized and listed also among the top recurrent dominant proteins in the selection with the sera from HP positive Healthy Controls.

As a second step, we looked at the domains/epitopes resulting as differentially enriched (*Q*-value (<0.05 and Focus (>0.8) in the selected phage libraries respect to the ORF-filtering genomic phage library; these were 115, 125, 183, and 117, respectively for the Healthy Control, Selection A, Selection B, and Selection C. As a third step among these domains we filtered out a sub-list of domains/epitopes specifically enriched only in the pathological outcomes and absent in the Healthy Control Selection. This step was aimed to reduce poly-reactive domains and consists of a subtractive comparison between the domains lists of the three pathological selections and the list of the Healthy Control Selection (Figure 2A).

Finally, we analyzed the overlap within the lists of domains/epitopes specifically enriched only in the Selection A, Selection B, and Selection C; as shown in Figure 2B in the Venn diagram most of the enriched domains, found after selections against sera (in total 138), are specific for the three pathological outcomes: Selection A (*n* = 25), Selection B (*n* = 85), and Selection C (*n* = 28), while only 45 enriched domains are common to two or to all the selections. In Supplementary Table 2 the complete list of the domains/epitopes specifically enriched in the selections is reported.

### Identification, Expression and Purification of a CagY/Cag7 Domain as Putative Antigenic Clone

**FIGURE 3:**
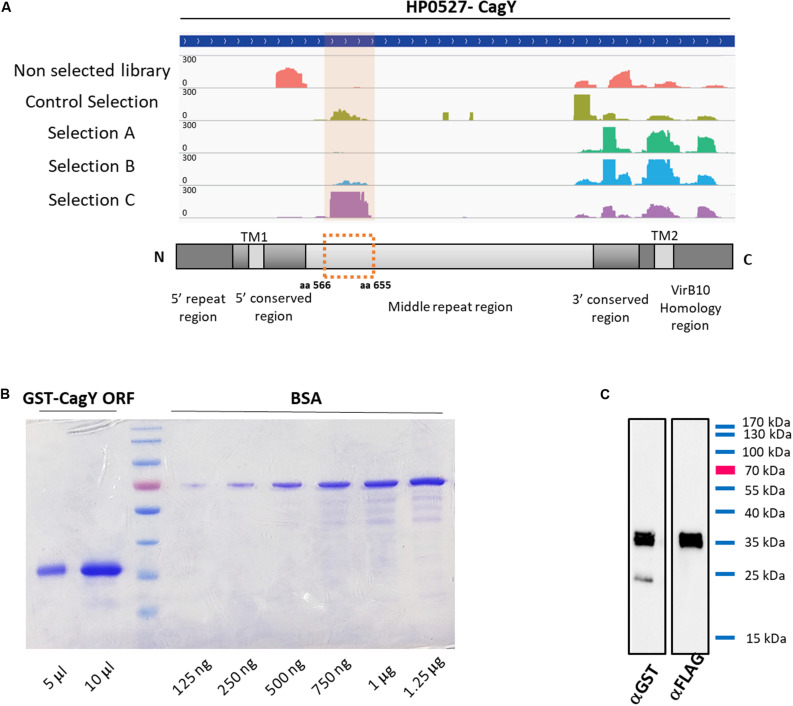
Characterization of selected CagY ORF. **(A)** Mapping of the reads for CagY on the reference gene sequence HP0527, before and after selection with patients’ sera and schematic representation of the CagY gene structure showing the alignment of the validated ORF on the CagY MRR domain. **(B)** Representative Coomassie gel staining of recombinant GST-CagY ORF-FLAG protein after purification on glutathione-coupled agarose resin, showing the absence of contaminants or degradation. BSA was used as a standard for protein quantification and a curve from 125 ng to 1.25 μg was run. **(C)** Representative western blotting of the purified GST-CagY ORF-FLAG protein with α-GST and α-FLAG antibodies, showing the integrity of the protein preparation.

As previously elucidated, it is very important to find novel biomarkers that can provide a “disease signature” for different pathological outcomes, or biomarkers associated to HP infection progression. Among the sub-lists of domains/epitopes specific for GC, AIG, MALT lymphoma, and absent in the HP + healthy controls we selected the top list ones, having the highest Fold Change values (see Supplementary Table 2) as putative candidate targets for production as recombinant protein and validation with ELISA assay. One of the most interesting domain enriched after sera selections was belonging to the gene HP0527, also named CagY/Cag7. The genomic library contains a large number of fragments mapping at the C terminal that were not specifically enriched after selection with sera. Interestingly after selection with sera of MALT lymphoma patients a domain mapping in their middle repeat region (MRR) was specifically enriched, mapping of the reads on the reference gene is shown in Figure 3A. HP0527 encodes for a large protein of 1927 amino acids described as one of the main components of *H. pylori* cag T4SS-associated pilum and proposed to be able to act as a sort of molecular switch that modifies the host pro-inflammatory responses by modulating the T4SS function and tuning CagA injection (Barrozo et al., 2013, 2016). To validate the antigenicity of this specific CagY domain, we recovered from the selected library the corresponding ORFs, thanks to two back-to-back oligonucleotides mapping to the center of the enriched domain. Several fragments of CagY protein were initially recovered from the library and finally the ORF portion corresponding to aa 566–655 of the CagY protein was chosen for subsequent validation. The selected ORF fragment was cloned in a vector allowing the production of a GST-ORF-FLAG recombinant protein. After purification by affinity chromatography the protein was checked by SDS-PAGE followed by Coomassie staining and showed more than 95% of purity (Figure 3B), Western blotting with both α-GST and α-FLAG antibodies (Figure 3C) confirmed the presence of full-length protein with very little degradation.

### Validation of CagY/Cag7 Protein Immunoreactivity by ELISA

**FIGURE 4:**
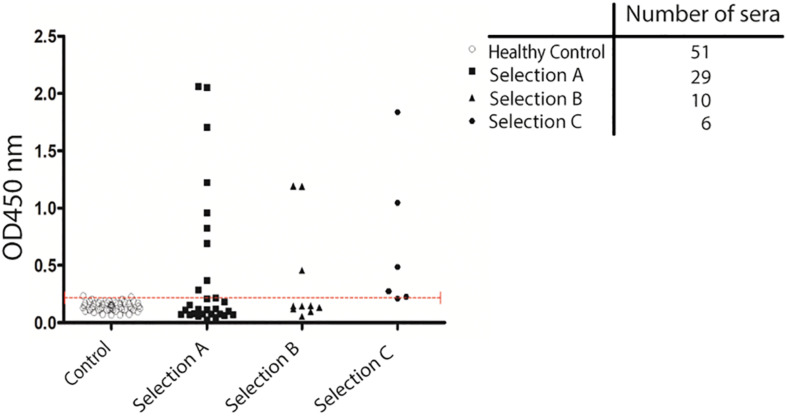
Validation of antigenicity of protein CagY by ELISA. Scatter plot showing the distribution of ELISA signal on CagY protein fragment. Antigen was challenged with a 1:500 dilution of sera from different groups of patients: HP positive and negative Healthy patients (51), from patients with Gastric Cancer (29 sera), Autoimmune Gastritis (10 sera), and MALT lymphoma (6 sera). Dotted line indicates cut-off level. Mann–Whitney U test was used to calculate differences between groups and a *p*-value < 0.05 was considered significant.

The recombinant purified protein was used as a capture antigen in an indirect ELISA. After preliminary set-up experiments at different antigen coating concentrations and sera dilutions, we established appropriate assay conditions. Finally, we performed a large-scale assay using all the recruited sera; the antigen was tested against a set of 96 serum samples obtained from different patients. Sera were collected from patients with GC (*n* (=29 sera), AIG (*n* = 10 sera), and MALT lymphoma (*n* = 6 sera). Fifty-one sera from healthy subjects both HP positive and negative were collected and used as controls. It should be noted that the number of patients affected by AIG and MALT lymphoma, that was possible to recruit, is much less than the number of patients affected by GC because these two pathologies are rare diseases. ELISA reactivity toward the purified CagY antigen was different among the tested groups (Supplementary Table 3). Cut off values for positivity was calculated using sera from healthy subjects that were showing globally a very low signal toward the antigen (mean OD = 0.142) and this reactivity was independent of the seropositivity for *H. pylori* infection (Supplementary Table 3). On the contrary, the specificity of the test was higher than 96% indicating that the protein domain tested is specific for infection progression toward pathological outcomes and not simply related to HP infection. Patients’ sera showed anti-CagY positive reaction, sensitivity was 31% of GC patients (9 over 29 sera), 30% of AIG patients (3 over 10 sera) and 83.3% for MALT Lymphoma patients (5 over 6 sera) (Figure 4). Comparison between sample groups showed a statistically significant difference in immunoreactivity of MALT lymphoma patients’ sera compared with control donors (*p* < 0.001, Mann–Whitney U test).

## Discussion

The mechanisms of GC induced by *H. pylori* are not fully understood. It is accepted that during the infection a complex and dynamic interaction between the bacterium and the host is established, in which the development of the disease is a result of both infecting *H. pylori* strain virulence and host immune set up. Accordingly, the identification and characterization of *H. pylori* proteins or protein domains able to evoke an immune response could be of great help to enhance understanding of how bacterium and host interact one with each other. The pathogen-specific humoral immune response is an important and informative host element that can potentially help to perform early and/or differential diagnosis allowing discrimination among disease severity.

In the last years many attempts to identify novel biomarkers for GC early diagnosis have been made and several studies have been published reporting the evaluation of antibody titer against different *H. pylori* antigens in different outcomes of infection In the majority of cases these antigens have been chosen based on previous evidence of their pathological role in *H. pylori* infection. Only a few studies reported an approach based on the use of the entire protein repertoire of the bacterium. In the most convincing study authors used immunoproteomic approaches and some *H. pylori* antigens were identified (Haas et al., 2002; Mini et al., 2006). Although this approach is unbiased it presents some drawbacks linked to the relatively high abundance of the antigen required by the assay and by the use of antigens in non-native conformations. Meinke et al. (2009) reported the use of a more comprehensive approach based on the bacterial display expression of *H. pylori* protein repertoire, and tried to define the antigenome profile of two *H. pylori* strains, recognized by patients with GC and duodenal ulcer. Although unbiased this approach has an important limit in the number of selected clones that can be analyzed by Sanger sequencing. Over the last years we have developed a high-throughput platform for the identification of novel biomarkers that takes advantage of next generation sequencing technology, which enormously increases the potentiality of the analysis. Our approach is based on the use of an innovative phage display system that using the ß-lactamase gene as an ORF and folding reporter allows to improve the functionality and the representativity of the proteins library fragments displayed on the surface of the phage. The platform has been validated in different contexts showing to be functional and useful for the identification of novel antigens in different pathological conditions and for the study of bacterial genomic DNA for advanced annotation (D’Angelo et al., 2011, 2013; Gourlay et al., 2015; Antony et al., 2019). Furthermore, in the context of bacterial-host interaction, further to be representative of the whole bacterial genomic DNA, our method allows also a representation of the complete ORFeome of a bacterium at the protein level, thus being a useful tool for the identification of novel biomarkers of the infection and/or of specific clinical outcomes. DNA typing has demonstrated that *H. pylori* strains are extremely different due to a high rate of mutation or recombination of their genome. This variability reflects strain virulence and may be paralleled by differences in protein expression or function (Owen, 1995). In order to obtain an antigenic repertoire of the bacterium as wider as possible, we used two *H. pylori* strains that were clinically isolated and whose genome sequences are completely available and fully annotated. Sequencing analysis of the phage libraries obtained showed that they were representative of the entire ORFeome of the bacterium, covering about 90% of the genome, thus the two phage libraries obtained were considered representative of the whole antigenic repertoire of *H. pylori*. Moreover, comparison of the two libraries both at the DNA and protein level showed that the nature of ORFs/domains present into both of them was extremely similar (Table 1) confirming the possibility of using them as a single reagent with no risk of introducing bias. After selection with patients’ sera antibodies the diversity of the libraries was significantly reduced, thus indicating that non-antigenic ORFs were lost during panning procedure and only potentially antigenic domains were selected. No bias toward one of the two HP strains was observed in the reads present in the selected libraries, indicating that the ORF repertoire of the two strains was equally represented on the phage particles.

Among the most enriched antigens recovered after selection we focused our attention on a domain of HP0527/CagY gene. This is an important component of the T4SS of the bacterium, and different reports have demonstrated that its presence is essential for *H. pylori* to induce cellular motility, adherence to target cell via α5β1 integrin binding, as well as CagA translocation and IL-8 induction (Rohde et al., 2003; Al-Ghoul et al., 2004; Koelblen et al., 2017). CagY is a surface-exposed protein of approximately 220 kDa protruding from the inner to the outer bacterial membrane (Kutter et al., 2008; Backert et al., 2015, 2016). The protein contains an unusual sequence structure with a high degree of variability given by the presence of two regions characterized by tandem repeats: a small 5′ repeat region (FRR) and a large MRR (Aras et al., 2003). Interestingly after selection, several clones, not perfectly identically mapping on the same region were recovered. The portion of the CagY selected corresponded to the N terminal part of the MRR region of the protein. These results indicate that the selection was done by virtue of a real antigenicity of the identified domain. The finding of antibodies against the MRR region of CagY is of particular interest since it has been demonstrated that this region controls the conformation of the full-length protein and, by modifying its integrin binding ability, is able to modulate T4SS function (Skoog et al., 2018); this mechanism is involved in the persistence of *H. pylori* infection. In-frame recombination events within the MRR repeats yield different CagY variants present in different bacterial strains. Moreover, recombination can also occur during infection, both *in vitro* and *in vivo* conferring to the bacteria both gain and loss of function. This finding led to the suggestion that this phenomenon is functional to immune evasion of the bacterium (Aras et al., 2003). However, the observation that CagY recombination occurs *in vivo* leading to the production of proteins with different size, both functional and non-functional, suggests that the bacterium could take advantage of inflammation at least in some situations. Recently Barrozo et al. (2013, 2016) demonstrated that CagY antigenic variation is not linked to the function of escaping the host immune response but rather to modulate it. Using both mouse and non-primate models they confirmed that CagY recombination is dependent to the host immune system pressure and showed that during infection it can both abrogate or restore the T4SS function. The authors propose that CagY works as a molecular switchable to “tune” the host immune response establishing the optimal inflammatory conditions to maximize the bacterium fitness. These findings highlight the hypothesis that CagY protein might be visible by the immune system at least in some phases of the infection and an immune response to it might be present in the host. Coherently the antigenicity of CagY has been demonstrated in rat and in mice were a *Lactococcus lactis* expressing the carbossi-terminal 383 amino acids has shown to induce immunity against CagY both at the mucosal and systemic levels (Kim et al., 2009). However so far a serum immune response to CagY has not been found in humans. Here, thanks to the use of an innovative approach we demonstrated that antibodies against CagY are present in the sera of individuals infected by *H. pylori* who have developed GC, AIG, and MALT lymphoma, thus relating immune response to CagY to HP infection progression toward pathological outcomes and in particular toward MALT lymphoma, as shown in Figure 4. Further investigations are needed, and larger sample size should be assayed in a more perspective way, to evaluate the feasibility of screening CagY antibodies as a potential biomarker in the diagnosis of *H. pylori* associated malignancies development.

To further investigate if the proteins’ domains significantly enriched with a log2 fold change higher that 1.5 and listed in Supplementary Table 2 have been already outlined as reactive antigens in previous literature we have overlapped these lists with the HP antigens list published by Meinke et al. (2009). The authors in this work have described the ANTIGENome of two clinical isolates of *H. pylori* derived from patients with GC and duodenal ulcer. By using 72 sera from patients with gastric adenocarcinoma and 324 HP positive controls, they identified 124 annotated HP genes and 54 non-annotated peptides as antigens. The 19 proteins that overlap between our list and the list published by Meinke et al. (2009) have been evidenced in yellow in Supplementary Table 2, worth of note is again CagY (Cag7) that in the list published by Meinke is the protein having the highest number of hits, even higher that the number of hits associated to CagA and VacA.

Among the proteins listed in Supplementary Table 2 we have focused our attention also on some other relevant hits that, in the light of previous literature, we have further discussed (these proteins are evidenced in bold in Supplementary Table 2). Among the proteins listed as uniquely recognized by sera from patients with GC (Selection A) and AIG (Selection B) we found the flagellar hook-associated protein FlgL (HP0295) and the flagellar basal body protein FliL (HP0809). It is known that *H. pylori* flagella may influence the colonization, inflammation, and immune evasion (Gu, 2017), in particular the hook junction protein FlgK and FlgL prevent the protein proteolysis when the flagellum is not assembled, while the gene coding for FliL is needed for swimming in pathogenic species of *H. pylori*. Finding antibodies selectively recognizing these proteins in the sera from infected patients can be considered relevant and promising for future validations. Another structural protein of the *H. pylori* flagellum that we find specifically enriched using sera of patients with AIG (Selection B) is the flagellar sheath adhesin HpaA (HP0797). This antigen is of particular interest since it has been demonstrated that is among the flagellar components expressed by *H. pylori* strains isolated from patients with stomach disease (Gu, 2017) and that it is a target of humoral immunity (Tang et al., 2008). In particular Tang and collaborators tested the positive rate of IgG antibodies against HpaA in the sera from 151 *H. pylori*-infected patients with chronic gastritis, peptic ulcer or gastric adenocarcinoma and found a target antigen positive/negative rate of 100% and a target antibody positive/negative rate of 87.4%. Thus, our finding of specific recognition of an HpaA protein domain by sera of patients with AIG can be considered relevant and promising for future validation of this antigen as a putative new biomarker also for this pathological outcome. We then found both in the list of proteins recognized by sera from patients with AIG (Selection B) and with MALT lymphoma (Selection C) some relevant outer membrane proteins (Voss et al., 2014) the iron-regulated outer membrane protein FrpB (HP0916–HP1512) and the iron(III) dicitrate transport protein FecA (HP1400). *H. pylori* needs iron in order to survive and in the human host there are several iron sources such as Transferrin (Tf), Lactoferrin (Lf), Hemoglobin (Hb), or haem. To obtain iron this pathogen has developed a mechanism consisting on expressing outer membrane proteins that bind Hb or haem (Braun and Hantke, 2011). Those proteins bind the iron source directly and several proteins have been investigated, for instance, FrpB is a protein family composed of three proteins: FrpB1, FrpB2, and FrpB3 (Dhaenens et al., 1997; Alm et al., 2000). *H. pylori* in particular is a human pathogen that requires a high concentration of iron because it has been reported that it can cause anemia when it is infecting humans (Beckett et al., 2016; Flores et al., 2017). This can explain why *H. pylori* has several genes, which express many proteins involved in iron acquisition such as FrpB1, FrpB2 or FrpB3, and FecA.

Both AIG and MALT lymphoma are known to be associated with anemia (Kulnigg-Dabsch, 2016; Pabona et al., 2016). AIG is also called pernicious anemia because one of the earliest symptoms of the disease is iron-deficiency anemia (IDA) which develops as a consequence of achlorhydria from oxyntic atrophy. During AIG the chronic inflammatory process causes, in the corpus and fundus of the stomach, a massive destruction of parietal cells, and this in turn determines a reduction of secretion of gastric acid required for the absorption of inorganic iron (Kulnigg-Dabsch, 2016) thus causing iron deficiency. *H. pylori* expresses FrpB1, FrpB2, and FrpB3 proteins to scavenge iron and they are regulated according to availability of iron source, in patients with AIG the iron is available in the stomach but it is not absorbed by the host, so it can be scavenged by *H. pylori*. Thus in the light of this work we think that our finding related to an antibody response against FrpB proteins in patients with AIG can be considered an important starting point for future validation of this protein also with sera from patients with AIG. At the same time we found enriched FrpB protein also in selection made with sera from patients with MALT lymphoma that is known to be associated with anemia as well. The mechanism in MALT lymphoma can be the same described for AIG, linking FrpB proteins expression to iron availability in the stomach of these patients.

A relevant increase was also found for the virulence-associated protein VapD (HP0967) by sera of patients with AIG (selection B). In a recent paper by Morales-Espinosa et al. (2020) high level of expression of virulence-associated protein D (VapD) of *H. pylori* were outlined inside adenocarcinoma gastric (AGS) cells and in gastric biopsies from patients suffering from different HP-related diseases. They observed that VapD is only expressed in the bacterium as a consequence of its interaction with the host cells, suggesting that it might contribute to *H. pylori* persistence inside the gastric epithelial cells, being involved in the protection of the bacterium from the intracellular environment and in its growth rate reduction, as well as enabling long-term infection and treatment resistance. However in their work Morales-Espinosa et al. (2020) Did not test the reactivity of patients sera against VapD protein, they analyzed VapD expression in AGS cells in *in vitro* time course experiments and they tested the expression level of VapD by RT qPCR in 82 gastric biopsies from patients outlying high expression levels of this gene/protein *in vivo* in particular in association with follicular gastritis. Thus, in the light of this work we think that our finding related to an antibody response against VapD in patients can be considered promising for future validations. Finally we found both in the list of proteins recognized by sera from patients with AIG (Selection B) and with MALT lymphoma (Selection C) the two homologs of the virulence factor CagE (Cag23), also annotated as protein VirB4 (HP0459–HP0544) (Shariq et al., 2015). This virulence factor has been previously found associated with HP-induced duodenal ulceration in children and enhanced production of IL8 that can impact the pathological outcome (Day et al., 2000), thus we think our finding of a specific antibody response against CagE in AIG and MALT can be considered again relevant an worth of future validations.

## Data Availability Statement

The datasets generated for this study can be found in the Sequence Read Archive Bioproject, Accession Number PRJNA506709.

## Ethics Statement

The studies involving human participants were reviewed and approved by the Careggi Hospital in Florence in collaboration with the Department of Experimental and Clinical Medicine of the University of Florence; Ethical committee protocol number 14936_bio. The patients/participants provided their written informed consent to participate in this study.

## Author Contributions

MS and GC performed the experiments and wrote the manuscript. SP performed the data analysis and wrote the manuscript. PE, MD’E, and CS participated to the experimental design and revised the manuscript. FC and AT collected samples and data from patients and contributed to the revision of the manuscript. DS and CP designed the experiments, wrote the manuscript, and supervised the research. All authors contributed to the article and approved the submitted version.

## Conflict of Interest

The authors declare that the research was conducted in the absence of any commercial or financial relationships that could be construed as a potential conflict of interest. The reviewer AD declared a past co-authorship with the author CP to the handling editor.

## Footnotes

1 https://imagej.nih.gov/ij/

## Supplementary Material

TABLE S1 List of all the domains/epitopes represented in the phage libraries obtained after selection against sera.

TABLE S2 List of the Differentially enriched domains/epitopes after selections with sera.

TABLE S3 List of the sera samples used for CagY domain validation and ELISA reactivity values toward the purified CagY antigen.
